# Dental unit waterlines in Israel: evidence of widespread microbial contamination and a need for national regulation

**DOI:** 10.1186/s13584-026-00778-9

**Published:** 2026-08-31

**Authors:** Luda Groisman, Eti Burla, Daniel Kushnir, Shereen Assaly, Roni Gat, Efrat Rorman, Shoshanit Ohad, Ehud Kaliner

**Affiliations:** 1 Public Health Division, Ministry of Health, Jerusalem, Israel; 2 Central District Bureau, Ministry of Health, Ramla, Israel; 3 Faculty of Health Sciences, Ben-Gurion University of the Negev, Beer-Sheva, Israel; 4 Soroka Clinical Research Center, Soroka University Medical Center, Beer-Sheva, Israel

**Keywords:** Microbial contamination, *Legionella* bacteria, Dental unit waterlines, Water quality, Regulatory guidelines

## Abstract

**Background:**

Dental unit waterlines (DUWLs) are susceptible to microbial contamination because of biofilm formation within their complex water delivery systems. Although the US CDC and several professional organizations recommend maintaining dental treatment water at potable water quality, Israel currently lacks specific national standards and regulatory requirements for monitoring and maintaining DUWLs. This study assessed the prevalence of *Legionella* and other microbial contaminants in DUWLs across dental clinics in central Israel and examined factors associated with contamination to inform patient and dental stuff safety and national policy development.

**Methods:**

This survey included 52 randomly selected dental clinics, with 359 water samples collected from DUWL components and water sources. Samples were tested for *Legionella* spp., *Legionella pneumophila*, *Pseudomonas aeruginosa*, and heterotrophic plate count (HPC). Water quality indicators - residual chlorine and turbidity were also measured. Statistical analyses were used to explore associations between microbial and common water quality and clinic characteristics.

**Results:**

Of the clinics tested, 94% exhibited at least one abnormal water quality result. *Legionella* exceeded the threshold in 74% of samples, *Pseudomonas* in 25%, and HPC in 53%. Contamination was most frequent in turbine and syringe connectors. Low chlorine levels and high turbidity were significantly associated with increased microbial counts. *Legionella pneumophila* concentrations above 2,500 GU/L were found in 19% of *Legionella*-positive samples. No consistent effect of dental chair age or clinic operating frequency was observed.

**Conclusions:**

The widespread microbial contamination identified in Israeli dental unit waterlines represents a potential patient and occupational health concern and highlights an important regulatory gap in the oversight of dental water quality. These findings support the need to develop national standards for DUWL monitoring, maintenance, and treatment, together with routine surveillance and regulatory oversight, to improve patient safety and ensure safe dental care environments.

**Supplementary Information:**

The online version contains supplementary material available at https://doi.org/10.1186/s13584-026-00778-9.

## Background

Safe water is an essential component of infection prevention and quality healthcare. Dental unit waterlines (DUWLs) are recognized as potential reservoirs of opportunistic waterborne pathogens because their design promotes biofilm formation and microbial proliferation. Although international organizations have issued recommendations for maintaining the microbiological quality of dental treatment water, regulatory approaches vary considerably between countries. In Israel, there are currently no specific national regulatory requirements governing the routine monitoring, microbiological quality, or maintenance of dental unit waterlines. This regulatory gap might have important implications for patient safety, occupational health, and quality assurance in dental care.

*Legionella* infections are reported worldwide, including in Israel, and are caused by bacteria of the genus *Legionella*. Infection typically occurs through inhalation of aerosolized water droplets containing the bacteria. Individuals at increased risk include adults over 50 years of age, males, smokers, and people with chronic lung disease, diabetes mellitus, chronic kidney disease, malignancy, or immunosuppressive conditions [1–4]. Clinically, infection may present as either Legionnaires’ disease, a severe form of pneumonia, or Pontiac fever, a milder influenza-like illness. *Legionella* bacteria are naturally present in aquatic environments but become a public health concern when they proliferate in inadequately maintained human-made water systems, where they colonize biofilms that protect them from environmental stress and disinfectants [5, 6].

Dental unit waterlines represent a particularly favorable environment for microbial growth. They consist of narrow-bore plastic tubing that supplies water to dental instruments such as high-speed handpieces, air-water syringes, and ultrasonic scalers. Their design and operation, including intermittent water flow, prolonged stagnation during periods of non-use, and a high surface-to-volume ratio within the tubing, facilitate biofilm formation and persistent microbial colonization [5, 7, 8].

Over the past four decades, numerous studies have demonstrated that DUWLs can become colonized by a wide variety of microorganisms, including bacteria, fungi, and protozoa [7].

DUWL biofilms are dominated by bacteria such as *Acinetobacter*, *Burkholderia*, *Legionella*, and *Pseudomonas*, which can cause opportunistic infections through aerosols generated during dental procedures [9–12]. In addition, non-tuberculous mycobacteria such as *Mycobacterium avium* and *M. chelonae*, fungi including *Candida* and *Aspergillus*, and protozoa like *Acanthamoeba* have also been detected, highlighting the complexity of DUWL microbiota and the potential health implications for both patients and dental personnel [13–15].

While documented cases of dental clinic-acquired Legionnaires’ disease, as well as other water-borne infections, appear rare, this likely reflects significant underreporting rather than true low incidence. In 2011 an 82-year-old woman in Italy developed fatal legionellosis two days after dental treatment, and molecular typing confirmed the clonal relationship between the clinical and environmental strains [8]. Similarly, an outbreak of *Mycobacterium abscessus* odontogenic infection was reported in children receiving treatment at a dental clinic in Anaheim, California [16].

However, the rarity of such documented cases contrasts sharply with serological evidence suggesting frequent exposure. Elevated levels of antibodies to *Legionella* have been found among dental professionals, including dentists, nurses, and hygienists, indicating regular occupational exposure that may not result in clinically recognized disease. Furthermore, dental healthcare workers have been reported to experience higher rates of respiratory infections compared to the general population and other healthcare professionals [17]. This discrepancy between rare documented cases and high antibody prevalence suggests that dental-associated *Legionella* infections are likely underdiagnosed due to nonspecific symptoms, delayed onset, and limited surveillance systems.

Several international organizations have published recommendations to minimize these risks. The United States Centers for Disease Control and Prevention (CDC) recommends that water used during routine dental treatment meet drinking water quality standards established by the United States Environmental Protection Agency (EPA), specifically containing fewer than 500 colony-forming units per milliliter (CFU/mL) of heterotrophic bacteria [18]. However, implementation and regulatory oversight differ considerably among countries, and many jurisdictions rely primarily on professional guidance rather than mandatory monitoring requirements [7].

In Israel, no specific national regulations govern the microbiological monitoring, maintenance, or control of water quality in dental unit waterlines unlike in other medical and healthcare facilities. The Oral Health Department of the Ministry of Health recommends flushing water and air through dental units at the start of each working day. National guidelines for the prevention and control of *Legionella* in water systems are mandatory for hospitals and healthcare facilities but remain advisory for other public institutions and businesses [19]. Importantly, these guidelines do not specifically address water quality in dental equipment or establish requirements for routine microbiological surveillance, validated disinfection protocols, or documentation of water quality management. Consequently, there is limited evidence regarding the microbiological quality of dental unit waterlines in Israel to inform national policy and regulatory decision-making.

Recent epidemiological investigations in Israel further highlight the need for such evidence. In the Central District alone, seven investigations of suspected *Legionella* exposure associated with dental treatment were conducted between 2021 and 2024, raising concerns regarding dental unit water systems as a potential source of waterborne pathogens.

The aim of this study was to assess the prevalence and distribution of *Legionella* and other microbial contaminants in dental unit waterlines in dental clinics across central Israel and to evaluate factors associated with bacterial contamination. To the best of our knowledge, this is the first systematic study of DUWL microbiological quality in Israel. The findings provide baseline evidence that may support the development of national standards for water quality monitoring, maintenance and disinfection protocols, and regulatory oversight to strengthen patient safety and infection prevention in dental care.

## Methods

### Study design

This cross-sectional study was conducted between February 7th, 2022 and February 18th, 2024 to assess microbiological contamination in dental unit waterlines across central Israel.

The participating clinics were randomly selected from a list of 228 dental clinics in central Israel registered by the Israel Ministry of Health (MoH), yielding a subsample of 52 clinics. This sampling approach ensured a representative geographic distribution, as described in the Results section. The selection criteria included geographic distribution across 17 localities in central Israel, clinic size, and operational frequency. Specifically, clinics were categorized as small (< 4 dental chairs) or large (> 4 dental chairs). For small clinics, one room was sampled, whereas for large clinics, two rooms were sampled.

Operational frequency was defined based on observed clinic activity patterns: private clinics typically operate 2–3 days per week for limited daily hours, whereas Health Maintenance Organization (HMO) clinics generally operate at least 5 days per week for 8–12 h per day. These differences may influence water stagnation in dental unit water lines and, consequently, the risk of microbiological contamination. The operational patterns were empirically validated. The final sample included 30 private clinics and 22 HMO-affiliated clinics from the three major health organizations in Israel (Clalit, Maccabi, and Meuhedet) ensuring representative coverage of the principal operational models within the Israeli dental healthcare system.

### Sample collection

Water samples were collected from each participating clinic during a single working day. Sampling included both input sources (tap water and distilled water reservoirs supplying the dental unit waterlines [DUWLs]) and output points (water from handpieces and mouthwash dispensers), in order to assess contamination throughout the entire dental unit water supply system.

All samples were collected in accordance with the Israel Ministry of Health (MoH) guidelines for water sampling and were transported to the laboratory under refrigerated conditions. Samples were processed within 6 h of collection to ensure sample integrity [20].

Common water quality parameters, including residual chlorine and turbidity, were measured on-site at each sampling point.

### Water analysis and regulatory thresholds

Detection and quantification of *Legionella* spp., *Legionella pneumophila*, and *L. pneumophila* serogroup 1 were performed using quantitative polymerase chain reaction (qPCR) in accordance with ISO/TS 12869:2019, employing the microproof^®^ *Legionella* Quantification LyoKit (BIOTECON Diagnostics) on a CFX96 Touch Real-Time PCR Detection System (Bio-Rad) [21]. To enable selective detection of viable bacteria, Reagent D (Hygiena^®^) was applied to suppress amplification of DNA from non-viable cells. *Pseudomonas aeruginosa* and heterotrophic plate counts (HPC) were determined according to Methods 9213E and 9215B, respectively, as described in APHA Standard Methods [22]. Turbidity and residual chlorine were measured on-site using a Hach 2100Q Portable Turbidimeter and a Hach Chlorine Test Kit CN-70, following the requirements of National Israeli Standard 6223 [23]. All analyses were conducted following standard quality assurance and quality control procedures required by the Israel Ministry of Health for drinking water testing.

Microbiological and physicochemical results were interpreted according to regulatory thresholds established by the Israel Ministry of Health (MoH). The threshold for *Legionella* spp. was set at ≥ 2,500 genomic units per liter (GU/L), and the same threshold was applied for *Legionella pneumophila* in the absence of a specific MoH guideline [19]. Notably, these interpretations rely on MoH-defined qPCR thresholds, which are calibrated for public health surveillance and are therefore not directly comparable to culture-based CFU/L values. For *Pseudomonas aeruginosa*, the threshold was ≥ 1 CFU/100 mL, and for HPC it was ≥ 200 CFU/mL. For physicochemical parameters, the acceptable range for residual chlorine concentration was ≥ 1 mg/L, and turbidity was expected to be less than 1 NTU [24]. Values exceeding these thresholds were considered abnormal and indicative of potential water quality issues.

If water samples failed to meet quality criteria, clinical operations were suspended immediately followed by comprehensive remediation and disinfection performed by specialized water treatment professionals. Systems were subsequently re-sampled to verify treatment efficacy; this cycle was repeated until standard quality limits were achieved. To ensure data integrity, these follow-up samples were excluded from the primary analysis (*n* = 359), which includes only the baseline results from each clinic’s initial sample.

### Data collection and statistical analysis

Clinic characteristics collected in the study included: clinic operational frequency as working days per week, dental chair age in categories of above or below 5 years, drinking water source (well or desalinated water).

Microbial culture counts, used for *Pseudomonas*, *Legionella* and HPC were described by proportions below or above predefined threshold out of available cases, geometric means (GM), along with the geometric mean standard deviation (GSD). Normally distributed variables were described using means and standard deviation (SD). We used Spearman correlation statistics to estimate the association between continuous microbial levels and other not normally distributed variables. We compared between subgroups using Chi-Square test for categorical variables, t-test for normally distributed continuous variables and ratio t-test – for microbial culture counts.

We modelled the microbial levels of *Pseudomonas*, *Legionella*, HPC, using water quality variables and clinics’ characteristics as potential risk factors. Clinics were defined as statistical clusters and their indicators were used as random effects in a mixed model regression. For each culture count two modelling approaches were used, (i) a logistic mixed model, where the outcome was categorized as above or below threshold and (ii) a negative binomial mixed model, where the outcome was presented as the absolute value of microbial count. The binary logistic model results were expressed as Odds Ratios (OR), indicating the increased risk of microbial levels exceeding their specific thresholds. The negative binomial model results were presented as a percent change in microbial levels corresponding to an increase of one unit in the explanatory variable. All independent covariates were checked for collinearity.

## Results

### Study population

The 52 sampled clinics provided a representative spatial coverage of the 228 dental clinics in central Israel. The clinics were located in 17 localities, with 57.7% (30/52) classified as private and 42.3% (22/52) belonging to Clalit, Maccabi, or Meuhedet Health Maintenance Organizations (HMOs). Between February 7, 2022, and February 18, 2024, a total of 359 water samples were collected from the 52 clinics, averaging 6.9 samples per clinic. 55% of dental chairs had been in use for more than five years and were classified as “old chairs.” Almost all clinics received municipal drinking water (well or desalinated) for their dental units; only two clinics used distilled water. Selected clinic characteristics are summarized in Table 1.

**Table 1:** Microbial culture count, water quality and clinic characteristics by clinic ownership

Characteristic	Overall, *N* = 359	HMO, *N* = 176	Private, *N* = 183	*p*-value
**Clinic Characteristics**				
Chair Age Above 5years, %(n)	55 (196)	55 (96)	55 (100)	0.998
Use frequency, % (n)				< 0.001
2–3 days/week	5 (19)	0 (0)	10 (19)	
6 days/week	95 (340)	100 (176)	90 (164)	
**Water Source**				
Well Water, % (n)	98 (353)	97 (170)	100 (183)	0.013
Desalination, % (n)	45 (161)	54 (95)	36 (66)	< 0.001
**Microbiological Water Quality**				
*Legionella*				
GM (GSD), GU/L	14,100 (11.2)	11,900 (13.3)	16,500 (9.3)	0.446
above threshold, % (n)	74 (267)	70 (123)	79 (144)	0.056
*Legionella pneumophila*				
GM (GSD), GU\L	315 (6.6)	399 (6.1)	251 (7.0)	0.002
*Pseudomonas aeruginosa*				
GM (GSD), CFU/100mL	0.065 (29.6)	0.132 (46.2)	0.034 (15.3)	< 0.001
above threshold, % (n)	25 (91)	33 (58)	18 (33)	0.001
*Heterotrophic Plate Counts*				
GM (GSD), CFU/100mL	130 (19.6)	122 (20.2)	139 (19.2)	0.788
above threshold, % (n)	53 (189)	56 (98)	50 (91)	0.258
**Physical-Chemical Water Quality**				
Residual Chlorine (mg/L)	0.16 (2.51)	0.15 (2.61)	0.16 (2.42)	0.882
No Residual Chlorine, % (n)	13.4 (48)	12.5 (22)	14.2 (26)	
Within 0.1–0.5 mg/L, % (n)	85 (305)	87 (153)	83 (152)	
Above 0.5 mg/L, % (n)	1.7 (6)	0.6 (1)	2.7 (5)	
Turbidity, NTU GM (GSD)	0.46 (2.60)	0.44 (2.62)	0.48 (2.59)	0.041
Above 1 NTU, % (n)	13 (45)	10 (16)	18 (29)	0.031
Temperature, ^o^C (mean ± SD)	23.7 ± 3.3	24.5 ± 3.2	22.8 ± 3.2	0.005

Of the 52 clinics tested, 49 did not meet one or more water quality criteria. Following initial treatment and repeated sampling, 12 of these 49 clinics still showed abnormal results and underwent additional water system treatments until acceptable water quality was achieved.

### Microbial count and water quality

The geometric mean and geometric standard deviation were 14,100 and 11.2 GU/L for *Legionella*, 315 and 6.6 GU/L for *Legionella pneumophila*, 0.065 and 29.6 CFU/100mL for *Pseudomonas*, 130 and 19.6 CFU/mL for HPC. In all, 74% (267/359) of samples tested above the required threshold for *Legionella*, 25% (91/259) were above the threshold for *Pseudomonas* and 53% (189/359) for the HPC. 85% (305/359) were within the required range for residual chlorine concentration and 87% (314/359) for turbidity. Distribution of microbial and and physico-chemical water quality parameters is shown in Supplementary Figs. 1, 2.

Further microbial counts are presented in Table 1, where they are also stratified by the clinics’ ownership. Chlorine levels were consistent between private and HMO clinics; however, turbidity exceeded the required threshold more often in private clinics (18% compared to 10%, p-value = 0.031). HMO clinics were open significantly more frequently than private clinics (< 0.001), consistent with differences in clinic activity patterns that may affect water stagnation and the associated risk of microbiological contamination. *Legionella* counts were more often above the threshold in private clinics compared to HMO clinics (79% vs. 70%) although this was of borderline significance (p-value = 0.056). Conversely, *Pseudomonas* counts exceeded the threshold more frequently in HMO clinics than in private clinics (33% vs. 18%, p-value = 0.001).

Spearman rank correlation analysis was performed to evaluate the associations between microbiological parameters.

A moderate to strong positive correlation was found between heterotrophic plate counts (HPC) and *Pseudomonas aeruginosa* concentrations (ρ = 0.57, *p* < 0.0001), indicating that samples with higher overall bacterial loads tended to exhibit higher levels of *P. aeruginosa.* In contrast, the correlations between HPC and *Legionella pneumophila* (ρ = 0.20, *p* < 0.0001) and between HPC and *Legionella spp*. (ρ = 0.19, *p* < 0.0001) were weak but statistically significant, suggesting only a limited association between general bacterial burden and the presence of *Legionella*.

A moderate positive correlation was observed between *Legionella spp*. and *Legionella pneumophila* (ρ = 0.43, *p* < 0.0001), consistent with the taxonomic relationship between the species and the genus. Conversely, no meaningful correlation was detected between *Pseudomonas aeruginosa* and *Legionella spp*. (ρ = −0.022, not significant). However, a weak but statistically significant positive correlation was identified between *Pseudomonas aeruginosa* and *Legionella pneumophila* (ρ = 0.14, *p* < 0.05).

**Fig. 1:**
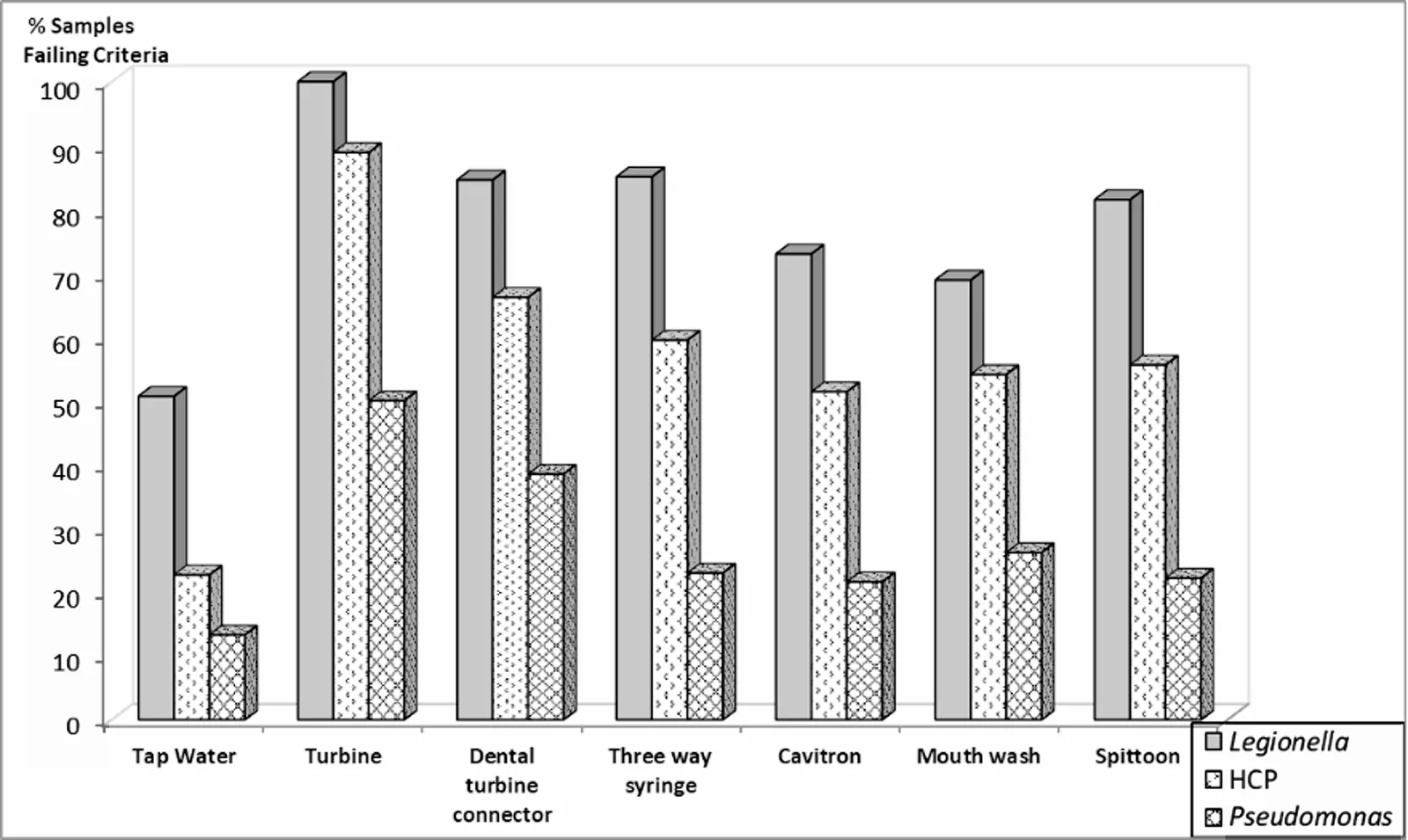
Prevalence of microorganisms in dental unit components exceeding water quality criteria

Microbial contamination levels varied notably across different dental unit water line sites (Table 2; Fig. 1). The turbine exhibited the highest contamination rates, with 100% of samples exceeding criteria for *Legionella*, approximately 90% for heterotrophic plate counts (HPC), and about 50% for *Pseudomonas*. Similarly, the dental turbine connector and three-way syringe showed high failure rates for *Legionella* (~ 85–90%) and HPC (60–70%), with moderate *Pseudomonas* contamination (25–40%). Cavitron, mouthwash, and spittoon samples demonstrated moderate contamination levels, with *Legionella* detection ranging from 70% to 85%, HPC between 50% and 60%, and *Pseudomonas* around 20–30%. Tap water samples had the lowest failure rates across all microorganisms (*Legionella* ~ 50%, HPC ~ 25%, *Pseudomonas* ~ 15%). No consistent correlations were observed between high counts of different microorganisms across sampling sites.

**Table 2:** Microbial culture count by collection site

Collection Site	*N* of samples	Legionella, GU/L		Legionella pneumophila, GU/L	Pseudomonas aeruginosa, CFU/100mL		Heterotrophic Plate Counts, CFU/100mL	
		> 2500 GU/L % (*n*)	GM (GSD)	GM (GSD)	> 1 CFU/100mL % (*n*)	GM (95%CI)^1^	> 200 CFU/100mL % (*n*)	GM ± GSD
Tap water	75	50.7% (38)	3000 (7. 8)	234 (4.8)	13.3% (10)	0.0258 (12.8)	22.7% (17)	16.7 (21.8)
Turbine	18	100% (18)	123000 (4.9)	704 (12.2)	50.0% (9)	0.311(42.7)	88.9% (16)	1390 (7.1)
Dental turbine connector	65	84.6% (55)	27500 (9.3)	390 (6.7)	38.5% (25)	0.168 (43.2)	66.2% (43)	295 (17.4)
Three way syringe	74	85.1% (63)	26,900 (9.8)	420 (7.4)	23.0% (17)	0.0683 (40.6)	59.5% (44)	286 (7.9)
Distilled water	2	100.0% (2)	70,400 (13.8)	153 (2.1)	0% (0)	0.0100 (1.0)	100% (2)	1080 (1.4)
Spittoon	27	81.5% (22)	13,800 (7.7)	228 (7.3)	22.2% (6)	0.0399 (16.4)	55.6% (15)	185 (14.6)
Cavitron	37	73.0% (27)	15,100 (11.1)	320 (7.9)	21.6% (8)	0.0514 (25.6)	51.4% (19)	89.0 (35.8)
Mouth wash	61	68.9% (42)	10,100 (12.2)	236 (5.69)	26.2% (16)	0.0707 (31.6)	54.1% (33)	130 (13.2)
All Sites	359	74.4% (267)	14,100 (11.2)	315 (6.65)	25.3% (91)	0.0656 (29.6)	52.6% (189)	130 (19.6)

^1^ Observations where the microbial count was 0, were converted to 0.01 for the calculations of geometric mean and standard deviation

It is noteworthy that in two clinics where distilled water from reservoirs was supplied to the dental unit water lines (DUWLs), high levels of *Legionella* and heterotrophic plate counts (HPC) were detected. Elevated microbial concentrations were also observed in the dental unit handpieces.

Two modifiable risk factors, Chlorine levels and turbidity, were found to impact the microbial levels in the multivariable logistic model (Table 3). This finding was consistent across the two types of modeling approaches, with binary and continuous microbial levels, although with varying amount of certainty. Specifically, Chlorine below the required threshold was associated with excessive levels of *Legionella* (> 2500GU/L) at OR = 13.2, although at borderline significance (95%CI: 0.98; 17.8, p-value = 0.002). This finding was confirmed by the negative binomial model showing that Chlorine levels below the required threshold are followed by an increase of 2.78% in the *Legionella* microbial counts (95%CI: 1.43; 5.38, p-value = 0.002). Similarly, turbidity was associated with increased levels of *Legionella* counts, whereas turbidity levels above threshold were associated with a change of 3.23% in the *Legionella* levels (95%CI: 1.84; 5.65, p-value < 0.001). This finding was partially confirmed by the logistic regression, which resulted in the same direction of the association but was not statistically significant.

**Table 3:** Factors associated with microbial culture count. Results of multivariable modeling

	Legionella				Pseudomonas aeruginosa				Heterotrophic Plate Counts			
	Logistic Mixed Model (> 2500 GU/l)		NB mixed model (continuous value)		Logistic Mixed Model (> 1 CFU/100 ml)		NB mixed model (continuous value)		Logistic Mixed Model (> 200 CFU/ml)		NB mixed model (continuous value)	
	OR	*p*-value	% change	*p*-value	OR	*p*-value	% change	*p*-value	OR	*p*-value	% change	*p*-value
Chlorine, < 0.1 mg/L	13.2	0.052	2.78	0.002	0.95	0.943	2.53	0.001	1.97	0.171	1.11	0.107
Turbidity, >1 NTU	2.47	0.281	3.23	< 0.001	2.59	0.085	0.45	0.001	2.26	0.074	1.07	0.237
Chair Age, > 5 year	1.80	0.271	9.52	0.821					1.27	0.472	1.06	0.188
Use frequency	5.58	0.168	0.81	0.107	1.20	0.637	1.24	0.369	0.87	0.537	0.96	0.211

Chlorine out of the required range was also a risk factor for the increased HPC, however this was not statistically confirmed judging by the significance levels. Higher turbidity was associated with higher HPC levels, however with varying significance across the modeling approaches.

Results of modeling *Pseudomonas* levels indicated an adverse impact of low Chlorine levels (2.53% change, p-value < 0.001 and 95%CI: 2.12; 3.30), however were inconsistent in terms of the impact of turbidity.

The impact of factors like dental chair age and the frequency of using the equipment was uncertain when adjusted to Chlorine and turbidity. No effect of water source (underground, desalinated) on the concentration of microorganisms in DUWLs was found.

## Discussion

Drinking water in Israel is strictly regulated by the Ministry of Health (MoH) and is routinely tested for chemical and microbiological parameters at the frequency defined in the drinking water regulations. Water that fails to meet these standards is prohibited from supply. Parameters such as *Pseudomonas*, heterotrophic plate counts (HPC), turbidity, and residual chlorine are included among the regulated criteria [25].

It is important to note that the quality of drinking water in public systems is the responsibility of the water supplier, and this responsibility extends only up to the entrance of the building. The regulations assume that buildings are equipped with water pipes and supply system components approved for use with drinking water in accordance with the Israeli National Standard N5452 [26]. Building owners are responsible for ensuring that the pipes under their ownership meet these standards. If owners have concerns about the condition or quality of their internal pipes, they may conduct additional water quality testing in their apartment or house by consulting the water supplier. These additional tests do not replace the regulatory monitoring performed by the MoH, but serve as a precautionary measure to ensure the safety of water within the building.

The absence of specific regulations for water quality in dental units in Israel means that safe water quality within these units cannot be fully assured, even though public drinking water is strictly regulated by the Ministry of Health (MoH). While the water supplied to buildings is routinely monitored for chemical and microbiological parameters, the internal water lines of dental units are susceptible to contamination due to conditions that promote microbial growth: small and narrow pipelines, intermittent water flow leading to stagnation and rapid biofilm formation, and inadequate maintenance of water lines and filters (both within the building and inside the dental units). Consequently, despite the overall quality of public drinking water, these factors create a high risk of microbiological contamination within dental unit water lines, which can compromise the safety of dental treatments.

In our study, it was found that 94% of the tested dental clinics the microbial quality of the water was abnormal. In 74% of the 359 tested water samples *Legionella* was found above the threshold level, 53% and 25% samples were abnormal for HPC and *Pseudomonas* accordingly. Similar results have been reported in research from different countries. *Legionella* was found in 71% of the water samples from 226 dental units and in 61% of the samples, HPC exceeded the recommended guidelines in the Netherlands [15]. 75% of 60 water samples collected from DUWL of private dental clinics and 87% of 30 samples from public clinics in Italy were contaminated with *Legionella*. Total viable count (TVC) was abnormal in 95% of the samples from private clinics and HPC was above the threshold value in 100% of the samples from public clinics. Concentration of *Pseudomonas aeruginosa* was abnormal in 17% of samples from public clinics [7, 27]. *Legionella pneumophila* was detected in 87% of 30 DUWL samples in Jordan [28].

Concentration of *Legionella pneumophila* above 2500GU/L (the threshold for abnormal value set in Israel for *Legionella* spp.) was detected in 51 samples from 16 dental clinics (19% from samples with abnormal results for *Legionella* spp.). This finding is worrying, our results are much higher than reports in developed countries: 6 and 29 *Legionella pneumophila* from 213 to 1377 samples with *Legionella spp*. in Netherlands and German respectively [15, 29].

*Pseudomonas aeruginosa* can lead to opportunistic infections causing devastating acute and chronic diseases in individuals with compromised immune systems. In addition, *P.aeruginosa* is a well-known biofilm former which effectively colonizes a variety of surfaces including medical materials and devices [30]. Aqueous biofilms in medical and dental devices can be a source of serious nosocomial infections. Biofilms offer a protective environment for microbial communities, pathogens not only survive but also multiply there. They are inherently resistant to biocides, and the biofilms in which they live increase their resistance [31].

HCP is included in guideline documents concerning water quality in dental units:

US CDC recommends that the level of dental unit waterlines be identical to the standards required for drinking water set by the US EPA and should be less than 500 colony-forming units per milliliter (CFU/mL) [32]. The American Dental Association has set a heterotrophic bacteria load of ≤ 200 CFU/mL for water delivered from dental unit waterlines [33]. In the EU, there is no current guideline for DUWLs, but in some countries reference is made to the drinking water standard or use their own recommendations [7]. For instance, according to the Royal Dutch Dental Association, water should meet criteria for HPC less than 100CFU/ml. If more than 10 ^4^ CFU /ml are present, an additional test for the presence of Legionella should take place [15].

There are differing opinions regarding the use of HPC/TVC as a measurement of water quality, especially as an indicator of *Legionella* contamination. The conclusion being that the cleanliness of DUWLs should be assessed by TVC this is because it is a good indicator of the presence of pathogens such as *Legionella* spp. and *P. aeruginosa* [34].

Other researchers found that while HPC provides a general indication of the water quality, it only supports the growth of 0.25% of the aquatic microorganisms [35] and is considered a poor indicator of *Legionella* spp. presence [36].

It should be acknowledged that according to our research, in 85 (32%) water samples from 267 with abnormal *Legionella* concentration including those with more than 10^5^GU/L, the HPC was below threshold value (< 200 CFU/mL). This finding casts doubt on whether HPC can be a reliable indicator of the presence of *Legionella* in DUWLs.

Water supplied to dental units usually contains less microorganisms than DUWLs water [27, 37]. As was mentioned above, drinking water is supplied directly from public supply to buildings where the clinics are located. The tested clinics did not have additional building-level water treatment systems, and as a result, microorganisms were detected in the tap water (51% *Legionella*, 13% *Pseudomonas*, 23% HCP), but these results are almost twice lower than in DUWLs. In 2 clinics distilled water from reservoirs was supplied to DUWLs. Although *Pseudomonas* was not found in this water, very high concentrations of *Legionella* and HPC were found. These findings highlight that, even when using ostensibly “clean” water sources such as distilled water, the conditions within the water containers and dental unit water lines—small-diameter tubing, intermittent flow, stagnation, and biofilm formation—can promote significant microbial contamination. This underscores the importance of proper maintenance and monitoring of DUWLs to ensure safe water for dental treatments. No effect of water source (underground, desalinated) on the concentration of microorganisms in DUWLs was found.

*Legionella* was the most prevalence organism (100%) in the turbine. *Pseudomonas* and HPC were also detected in turbine in higher concentrations, then in other DU parts. In other parts occurrences of measured microorganisms were similar (*Legionella* 70–85%, *Pseudomonas* 23–26%, HPC 51–59%). As discussed earlier, DUWL are densely populated with microorganism resulting from supplied water (which is usually free of pathogens, but not sterile) and by suck-back of biological liquids from the oral cavities of the patients [7]. Water transported through narrow pipes, valves and connectors, laminar flow and stagnation while dental unit is not in use promote biofilm formation. Microorganisms in water will form after their adhesion, a matrix-covered biofilm capable of withstanding a variety of external stresses such as antimicrobial treatment. When biofilm forms on all surfaces of the DUWS, check valves begin to fail thereby human liquids can be sucked back into the DUWLs [15].

This study revealed distinct contamination patterns between private and HMO clinics. Despite similar chlorine levels, private clinics showed higher turbidity and a tendency toward increased *Legionella* contamination, whereas *Pseudomonas* was significantly more frequent in HMO clinics. These patterns could be related to clinic operating frequency, raising the possibility that differences in water usage influence contamination profiles: *Legionella* proliferation is often linked to prolonged water stagnation, while *Pseudomonas* thrives in biofilm-rich systems sustained by continuous water flow and nutrient supply [38].

However, in our study we did not observe a clear impact of usage frequency and chair age on microbial contamination of DUWLs or on physical–chemical water quality.

Residual chlorine concentration and turbidity correlated, as expected, with microbiological water quality. High turbidity values and absence of chlorine residue positively correlated with abnormal values of microbiological parameters.

The study has several limitations. First of all, the study does not address the clinical significance of the findings. Second, the study does not compare the findings to exposure in our usual environments (domestic or otherwise) and whether there are significant differences between them. In addition, the study has potential biases – sampling at different times of the year with varying climates in different locations and various professionals sampling the water.

Another limitation is the exclusive use of qPCR-based detection for *Legionella*. Although a viability treatment (Reagent D) was applied to reduce detection of non-viable organisms, genome unit measurements (GU/L) are not directly equivalent to culture-based CFU/L counts, and their relationship remains variable. However, this approach aligns with the Israel Ministry of Health guidelines, which are based on national validation studies that established calibrated qPCR thresholds for public health decision-making. Accordingly, these findings should be interpreted within validated qPCR-based frameworks, which provide a standardized and policy-relevant basis for assessment, while caution is warranted when comparing results with culture-based studies.

## Conclusions

This study demonstrates widespread microbial contamination of dental unit waterlines in dental clinics in central Israel, with the vast majority of clinics exhibiting at least one abnormal microbiological water quality result. The findings indicate that contaminated DUWLs may represent a preventable risk to both patients and dental healthcare personnel, particularly through exposure to aerosolized water during routine dental procedures.

The study also highlights an important regulatory gap. While the Israeli Ministry of Health has established guidelines for the prevention and control of *Legionella* in healthcare facilities and other public water systems, there are currently no specific national requirements addressing the monitoring, maintenance, and microbiological quality of dental unit waterlines. The high prevalence of contamination observed in this study suggests that existing recommendations are insufficient to ensure consistent water quality in dental practice.

As the first systematic assessment of DUWL microbiological quality in Israel, this study provides evidence that can support the development of national standards for dental water quality. Such standards should include routine microbiological monitoring, validated maintenance and disinfection protocols, documentation of water quality management, and appropriate regulatory oversight. Strengthening these measures would contribute to improved infection prevention, enhanced patient and occupational safety, and higher quality dental care.

### Recommendation

We recommend that the Ministry of Health develop and implement national guidelines for the routine monitoring, maintenance, and treatment of dental unit waterlines, together with an appropriate regulatory framework to ensure compliance and protect public health.

## Supplementary Information

Below is the link to the electronic supplementary material.


Supplementary Material 1: Additional file 1: Supplementary Fig1: Distribution of microbial count in the study population (359 water samples); Supplementary Fig2: Distribution of water quality parameters in the study population (359 water samples)


## Data Availability

The data underlying this article cannot be shared publicly in order to protect privacy of study participants. The anonymized data will be shared upon reasonable request to the corresponding author.

## Abbreviations

CFU: Colony-forming units
DUWLs: Dental unit waterlines
GM: Geometric means
GSD: Geometric mean standard deviation
HMO: Health Maintenance Organizations
HPC: Heterotrophic plate count
MoH: Ministry of Health
NTU: Nephelometric Turbidity Units
OR: Odds Ratios
SD: Standard deviation
TVC: Total viable count
US CDC: United States Centers for Disease Control and Prevention
US EPA: United States Environmental Protection Agency
