# Supplementary material for: Dental unit waterlines in Israel: evidence of widespread microbial contamination and a need for national regulation
Source: Isr J Health Policy Res. 2026 Aug 31;15:34. doi: 10.1186/s13584-026-00778-9 (PMC13528164; doi:10.1186/s13584-026-00778-9)
Supplement: Supplementary file 1 [file 13584_2026_778_MOESM1_ESM.docx]

**Supplementary Materials**

**Supplementary Fig 1**. Distribution of microbial count in the study population (359 water samples)

**Supplementary Fig 2**. Distribution of water quality parameters in the study population (359 water samples)
